# Quality of Life, Psychological Wellbeing, and Sexuality in Women with Urinary Incontinence—Where Are We Now: A Narrative Review

**DOI:** 10.3390/medicina58040525

**Published:** 2022-04-09

**Authors:** Matteo Frigerio, Marta Barba, Alice Cola, Andrea Braga, Angela Celardo, Gaetano Maria Munno, Maria Teresa Schettino, Primo Vagnetti, Fulvio De Simone, Alessandra Di Lucia, Giulia Grassini, Marco Torella

**Affiliations:** 1 Gynecology Department, San Gerardo Hospital, Milano Bicocca University, ASST Monza—Via Pergolesi 33, 20900 Monza, Italy; m.barba8792@gmail.com (M.B.); alice.cola1@gmail.com (A.C.); 2 Ospedale Regionale di Mendrisio Beata Vergine, 6850 Mendrisio, Switzerland; andrea.braga@eoc.ch; 3 Gynecology Department, Campania Luigi Vanvitelli University, 81100 Naples, Italy; angelacelardo@gmail.com (A.C.); gmm9401@gmail.com (G.M.M.); mariateresa.sche@libero.it (M.T.S.); primo.vagnetti@umicampania.it (P.V.); fulviodesimone65@gmail.com (F.D.S.); aledilucia@gmail.com (A.D.L.); grassini9@gmail.com (G.G.); marcotorella@iol.it (M.T.)

**Keywords:** sexual function, urinary incontinence, urge-urinary incontinece, stress urinary incontinence, mixed urinary incontinence, coital urinary incontinence, review, sexuality

## Abstract

Urinary incontinence (UI) is a very common condition, negatively affecting social, occupational, domestic, and psychophysical wellbeing. In particular, a peculiar and detrimental effect of UI has been described concerning sexual function. However, the impact of UI on quality of life is not fully understood yet, and further investigation into this issue is warranted. With this narrative review, we aimed to report the current evidence from recent literature regarding the quality of life and psychological wellbeing in patients with urinary incontinence, with a special focus on sexual function and its evolution after UI treatment. There is strong evidence that urinary incontinence—in its different forms, including stress urinary incontinence, urge urinary incontinence, mixed urinary incontinence, and coital urinary incontinence—negatively affects female sexual function. Treatments aimed to cure urinary incontinence—including pelvic floor muscles training, medications, and surgery—seem to improve quality of life by recovering, at least in part, sexual function. In conclusion, there is a substantial association between involuntary urinary loss and sex life quality. However, few studies are available and more evidence is needed before consistent conclusions can be made.

## 1. Introduction

Urinary incontinence (UI) is defined as the complaint of any involuntary loss of urine [1]. It is a very common condition, affecting the female population with a prevalence ranging from 15% to 55% [2]. It negatively affects social, occupational, domestic, and psychophysical wellbeing [3]. In particular, a peculiar and detrimental effect of UI has been described concerning sexual function. This has been estimated to occur in 26% of women with stress urinary incontinence (SUI) and 43% of women with urge urinary incontinence (UUI) [4]. Specifically, diminished libido, vaginal dryness, and dyspareunia have been described irrespective of age, educational level, and race in women with urinary incontinence [5]. Moreover, a peculiar aspect of incontinence-related sexual dysfunction is represented by coital incontinence. This may affect up to 25% of women attending a urogynaecological clinic and can be either associated with leakage on penetration or orgasm [6]. In addition, UI may condition the avoidance of sexual intercourses. It has been reported that sexual inactivity increases from 35% to 50% in women with UUI compared to controls, and this seems to be directly associated with incontinence severity [7,8]. Lastly, a negative impact has also been demonstrated on the sexual function of male partners of women with UI in terms of less satisfaction and more erectile dysfunction compared with controls [9].

However, the impact of UI on quality of life is not fully understood yet, and further investigation into this issue is warranted. Available reports use different definitions and analyze the impact of various treatments on different outcomes measures, thus making it difficult to develop a comprehensive overview of this issue. With this narrative review, we aimed to report the current evidence from recent literature regarding the quality of life and psychological wellbeing in patients with urinary incontinence, with a special focus on sexual function and its evolution after UI treatment.

## 2. Stress Urinary Incontinence

Stress urinary incontinence is defined as urinary leakage in the presence of an increase in intra-abdominal pressure without detrusor activation [10]. It is caused by urethral intrinsic deficiency, loss of urethral supports, or both. Conservative treatment of SUI involves the strengthening and re-education of pelvic floor muscles through physical exercises of pelvic floor muscles, biofeedback, and electro-stimulation techniques. Surgical treatment is managed through the injection of submucosal polymers around the sphincter, sub-urethral slings, or Burch colposuspension. SUI has been shown to negatively affect female sexual function in terms of less frequent sexual activity, less satisfaction, and more avoidance of sex compared to women without lower urinary tract symptoms (LUTS) [11]. When the impact of SUI on sexuality is compared with other forms of urinary incontinence—such as UUI or mixed urinary incontinence (MUI)—evidence is conflicting. While SUI had a smaller effect on sexual function than UUI or MUI in most studies, a Turkish study on 111 women showed that patients with urodynamic SUI had significantly lower Pelvic Organ Prolapse/Urinary Incontinence Sexual Questionnaire (PISQ-12) scores (meaning a higher sexual function impairment) compared to patients with UUI (*p* = 0.015) [2,12]. Similarly, in a Swedish cross-sectional study on 147 sexually active women with UI or overactive bladder (OAB), there was a tendency that women with SUI were more unsatisfied with their sexual life than those with MUI or OAB [13]. Specifically, in the SUI group, a negative impact on sexual desire and/or sexual satisfaction for the SUI group was found regarding insufficient vaginal lubrication, unsatisfying partner relationships, difficulties in reaching orgasm, and worries about urinary leakage during sexual activity. Other studies reported that some sexual disorders are specifically related to SUI compared to other forms of urinary incontinence, such as dyspareunia and coital urinary incontinence [14,15]. In particular, the latter seems to be associated with urethral dysfunction [16].

Literature suggests that successful treatment of SUI can improve overall female sexual function scores. Specifically, non-surgical management through pelvic floor muscle training (PFMT) seems to be associated with an improvement in sexual life [17]. Using validated tools, namely the Quality of Life Scale (QoLS-N) and the Bristol Female Lower Urinary Tract Symptoms (B-FLUTS) questionnaire, Bø et al. demonstrated a significant reduction in sex life, social life, and physical activity-related issues after 6 months of PFMT compared to controls [18]. Similarly, Zahariou et al. reported an improvement in all domains and total score of the Female Sexual Function Index (FSFI) after a 12-month program of supervised PFMT in a cohort of 70 women [19]. Moreover, surgical management of SUI seems to have a positive impact on sexual function according to reports. Overall, a certain grade in the improvement in sexual function has been described for all surgical procedures—in terms of less restriction of sexual activity as a result of fear of incontinence or negative emotional reactions during sex—while cases of deterioration related to de novo dyspareunia have been associated with concomitant procedures, such as posterior colporrhaphy [8,20]. The mechanism through which anti-incontinence surgery is believed to improve sexual function is the cessation of SUI, in particular when this is associated with coital incontinence [21,22]. No substantial differences in the impact of different procedures on sex life have been uncovered in comparison studies. Ward and Hilton assessed sex life in a 5-year extension of a multicenter randomized controlled trial of midurethral sling versus Burch colposuspension, and found no differences between groups [23]. Similarly, different types of tape do not seem to differently affect the impact on sexual function. Specifically, available studies comparing retropubic (TVT) and transobturator (TOT) approaches yield similar results on sex function [24,25,26]. Jha et al. evaluated 113 women with pure SUI who underwent either TVT or TOT tapes, and who completed the PISQ-12 preoperatively and 6 months after the procedure [24]. The postoperative PISQ-12 score improved in both the physical and partner-related domains, and a reduction in episodes of coital incontinence was observed, irrespective of the surgical approach (retropubic or transobturator) performed. Similarly, other findings, reporting either the PISQ-12 or the Female Sexual Function Index (FSFI) postoperative scores, did not find significant differences in sexual function between the transobturator and the retropubic surgical routes [25,26]. This seems to be also true for single-incision slings when compared to either retropubic or transobturator tapes, confirming that the positive impact of suburethral tapes on sexual function is likely to be independent of the type of sling [27,28]. Very little data are available about sexual function after urethral bulking agents. This may also be due, at least in part, to the fact that—in most settings—this procedure is often reserved for patients ‘unfit’ for other anti-incontinence surgical procedures, due to multiple comorbidities. Consequently, assessment of sexual function in these women may be difficult due to the presence of several confounding variables. A randomized trial comparing TVT (111 patients) and urethral bulking agent (113 patients) for SUI treatment showed similar improvement in sexual function according to the PISQ-12 total scores [29]. Similarly, postoperative dyspareunia remained unchanged in both groups and there was no difference between the groups after 1 year. Recently, energy-based treatments have been proposed for the treatment of stress urinary incontinence. Desai et al. reported an improvement in both stress urinary incontinence and sexual function according to FSFI scores in a cohort of 37 women with SUI after radiofrequency treatment [30]. Similarly, Kuszka et al. demonstrated an improvement in PISQ-12 scores in a population of 59 women with SUI treated with an Erbium YAG laser [31].

## 3. Urge Urinary Incontinence

Urge urinary incontinence is the involuntary loss of urine in correspondence with detrusor activation and represents a symptom of overactive bladder (OAB) [10]. OAB is a condition with a great impact on quality of life, including physical, social, psychological, and sexual aspects. It may be associated with neurological lesions or idiopathic, with the latter representing the predominant form. Urodynamic studies may—or may not—demonstrate detrusor hyperactivity in these patients. OAB and/or UUI treatment is aimed to relieve symptoms, and includes behavioral therapy, PFMT, and pharmacological treatment (antimuscarinic and ß3-agonists). Second-line treatments involve intradetrusor injections of botulinum toxin, neuromodulation (through posterior tibial nerve stimulation or sacral modulation), and exceptionally bladder augmentation enterocystoplasty [32,33]. Studies clearly show that UUI has a negative impact on sexual function. A Korean cross-sectional study investigating LUTS and sexual activity in 3372 premenopausal women with a structured internet survey found that those with OAB spectrum had a 4.8 times higher risk of sexual impairment compared to asymptomatic women [34]. Interestingly, patients with UUI have worse sexual function, even compared to women with other LUTS, such as SUI, in particular concerning sexual activity, orgasmic problems, and dyspareunia [4,12,35].

Less evidence is available about the impact of different OAB treatments on sex life. Specifically, anticholinergic medications have been shown to improve the overall quality of life, but data on their effect on female sexual function are scarce. Oral therapy was evaluated in two non-randomized studies by Zachariou et al., comparing women with OAB treated with pharmacologic therapy—either extended-release tolterodine 4 mg or mirabegron 50 mg—to subjects who refused any treatment [36,37]. In both papers, pharmacological treatment demonstrated an improvement in sexual function according to FSFI scores. Similarly, there is little published on the effect of second-line treatments for refractory OAB on female sexual function. Intradetrusor injections of onabotulinum toxin A 100 U was evaluated in two prospective studies on an idiopathic population with UUI [38,39].

In both reports, an improvement in sexual function according to FSFI scores was noted, although their study populations were limited. Lastly, in a small study of 11 sexually active patients who underwent sacral neuromodulator implantation for OAB, an increase in desire, lubrication, orgasm, satisfaction, pain, and global sexual function was observed according to FSFI. Interestingly, no correlation was found between FSFI scores and relief of urinary symptoms [40].

## 4. Mixed Urinary Incontinence

Mixed urinary incontinence (MUI) is defined as the coexistence of both SUI and UUI [10]. Most studies—unsurprisingly—suggest that the impact of MUI on sexual function is worse than the one observed in the case of isolated SUI or UUI [11]. An Israeli cross-sectional study on 187 women demonstrated worse sexual function according to the PISQ-12 questionnaire in women with MUI compared to patients with isolated SUI [41]. Similarly, a Turkish cross-sectional study on 118 sexually active women observed lower mean PISQ-12 scores in MUI patients compared to those with isolated SUI and UUI [42]. From a qualitative point of view, a greater detrimental impact on sexual function is reported in patients with MUI compared to SUI, UUI, and continent women, for all FSFI domains—namely desire, arousal, lubrication, orgasm, satisfaction, and pain [43].

Since MUI encapsulates different entities—such as UUI-predominant and SUI-predominant MUI—treatments can be very heterogeneous and data can be difficult to analyze, with particular reference to the impact on sexual function. However, the impact of UI treatment strategies on sex life is reported in corresponding paragraphs.

## 5. Coital Urinary Incontinence

Coital urinary incontinence (CUI) is defined as the “complaint of involuntary loss of urine during coitus” [10]. This is very likely to be the most under-reported form of UI due to embarrassment. In particular, it seems that women very seldom voluntarily consult on the issue of coital incontinence unless they are asked directly by physicians or self-compiled questionnaires [15]. Consequently—compared to other types of UI—coital incontinence may be particularly difficult to understand and research. According to a systematic review, this represents a frequent condition with prevalences ranging between 10% and 27% [22]. However, for women attending urogynecology clinics for symptomatic urinary incontinence, the prevalence may be much higher—up to 56%—but less than a tenth of them seek consultation for this disorder before direct questioning [16]. Due to the limited data, the pathophysiology of coital incontinence is not fully understood. Traditionally, it is divided into incontinence during penetration and incontinence at orgasm. The first is believed to be more prevalent in women with stress incontinence, while the latter is considered to be more common in women with detrusor overactivity. Overall, CUI seems to be more prevalent in patients with urodynamic stress incontinence compared to detrusor overactivity, and urethral dysfunction has been proposed as the possible causative mechanism [15]. In particular, low maximal urethral closure pressure was found to be a four-times fold the independent predictor of CUI in an observational study involving 505 sexually active women [16]. Although urinary incontinence during coitus may obviously lead to reduced sexual desire, reduced ability to achieve an orgasm, and impaired sexual relationships, its impact on quality of life is still unclear due to difficulties in investigating this condition [44]. Recently, the International Female Coital Incontinence Questionnaire (IFCI-Q)—specifically designed to evaluate the presence, severity, and type of CUI and its impact on the quality of sexual intercourse—has been developed and validated [45]. Authors found that CUI restricts sexual activity in 57% of patients, negatively interfered with achieving orgasm in 67% of them, and up to 77% would abstain from sexual activity. These data are in line with previous reports that confirmed a significant impact of CUI on patients’ quality and frequency of sexual activity [6]. Gray et al., in 2017, evaluated a total of 2312 women using the electronic Personal Assessment Questionnaire Pelvic Floor (ePAQ-PF) in advance of their urogynecology consultation. They found that in women with CUI, there was significant self-avoidance of sex, partner avoidance of sex, and impaired quality of sex life due to sexual problems compared to controls [46]. Interestingly, in a subgroup of patients, midurethral sling surgical treatment resulted in a significant improvement in all coital incontinence symptoms 3 months postoperatively [46]. CUI has been shown to negatively affect the overall quality of life according to the King’s Health questionnaire in all domains apart from sleep/energy [47]. A detrimental impact on psychological wellbeing has also been described in women with CUI. In a cross-sectional Swedish study, half of the women with CUI said that their sexual life was spoiled from worry, and dissatisfaction with sexual life was strongly correlated to unsatisfying psychological health [13]. In another study, 80% of patients said they feel depressed due to CUI [45]. However, data on the impact of CUI on psychological health are limited, and further studies that use validated tools are advisable before making conclusions.

The few studies available regarding the effects of PFMT on female sexual function in patients with CUI report a significant improvement in women’s sexuality as a consequence of a reduction in the episodes of incontinence during intercourses [18,19]. Other treatments for CUI are usually tailored on urodynamic findings and may refer to either SUI or UUI usual management strategies. In women complaining of CUI during orgasm with documented detrusor overactivity, antimuscarinic treatment resulted in symptomatic relief in 60% of patients [21]. In women with CUI on penetration and urodynamic SUI, up to 80% are successfully treated with anti-incontinence surgery [22].

## 6. Conclusions

The current review summarizes the impact of urinary incontinence on sexual function. There is strong evidence that urinary incontinence—in its different forms—negatively affects female sexual function. Treatments aimed to cure urinary incontinence—including pelvic floor muscles training, medications, and surgery—seem to improve quality of life by recovering at least in part sexual function. Since this was not a systematic review, a possible limitation may be found in the narrative nature of this work; thus, some studies containing important information have not been retrieved and analyzed as a consequence of the searching strategy. However, more evidence is needed before consistent conclusions can be made.

## Data Availability

Not applicable.
